# Understanding Large Language Models in Healthcare: A Guide to Clinical Implementation and Interpreting Publications

**DOI:** 10.7759/cureus.82397

**Published:** 2025-04-16

**Authors:** Julia Maslinski, Rachel Grasfield, Raghav Awasthi, Shreya Mishra, Dwarikanath Mahapatra, Piyush Mathur

**Affiliations:** 1 Artificial Intelligence, BrainXAI ReSearch, BrainX LLC, Cleveland, USA; 2 Integrative Neuroscience, Binghamton University, Binghamton, USA; 3 Medicine and Health Sciences, University of Iowa, Iowa City, USA; 4 Population and Quantitative Health Sciences, Case Western Reserve University, Cleveland, USA; 5 Data Science, Lerner Research Institute, Cleveland Clinic, Cleveland, USA; 6 Anesthesiology and Perioperative Medicine, Cleveland Clinic, Cleveland, USA; 7 Anesthesiology, Case Western Reserve University School of Medicine, Cleveland, USA

**Keywords:** artificial intelligence in medicine, deep learning artificial intelligence, health professional's education, large language models (llms), large language models (llms) in medicine, research

## Abstract

Large language models (LLMs) have generated excitement and interest in their capability to impact various facets of healthcare delivery. However, the rapidly expanding literature on LLMs presents challenges in understanding recent work, associated terminology, and potential applications for healthcare professionals. In this review, we discuss the development and evolution of LLMs, especially in healthcare. We provide a description of the key terminologies associated with LLMs to improve the understanding of these terms and their application context in healthcare. Evaluation of the experiments and research related to LLMs is fundamentally important for clinicians. Thus, we provide a description of evaluation methodologies used in LLM research. Lastly, through illustrative examples of research in application of LLMs in healthcare, we showcase the opportunities to leverage this state-of-the-art artificial intelligence (AI) technique with considerations for clinical and administrative adoption both by the patients and healthcare professionals. Through this review, we hope to equip the healthcare professionals with the knowledge they need to understand LLM in healthcare research.

## Introduction and background

The number of publications related to artificial intelligence (AI) in healthcare using large language models (LLMs) is increasing across all aspects of healthcare [1-4]. This is further substantiated by the emergence of journals solely dedicated to AI in healthcare, which actively support research and publications related to LLMs in healthcare [5]. We have observed a similar ‘infodemic’ for image-based AI in healthcare research using deep learning in the fields of radiology, gastroenterology, and cardiology [6].

**Figure 1:**
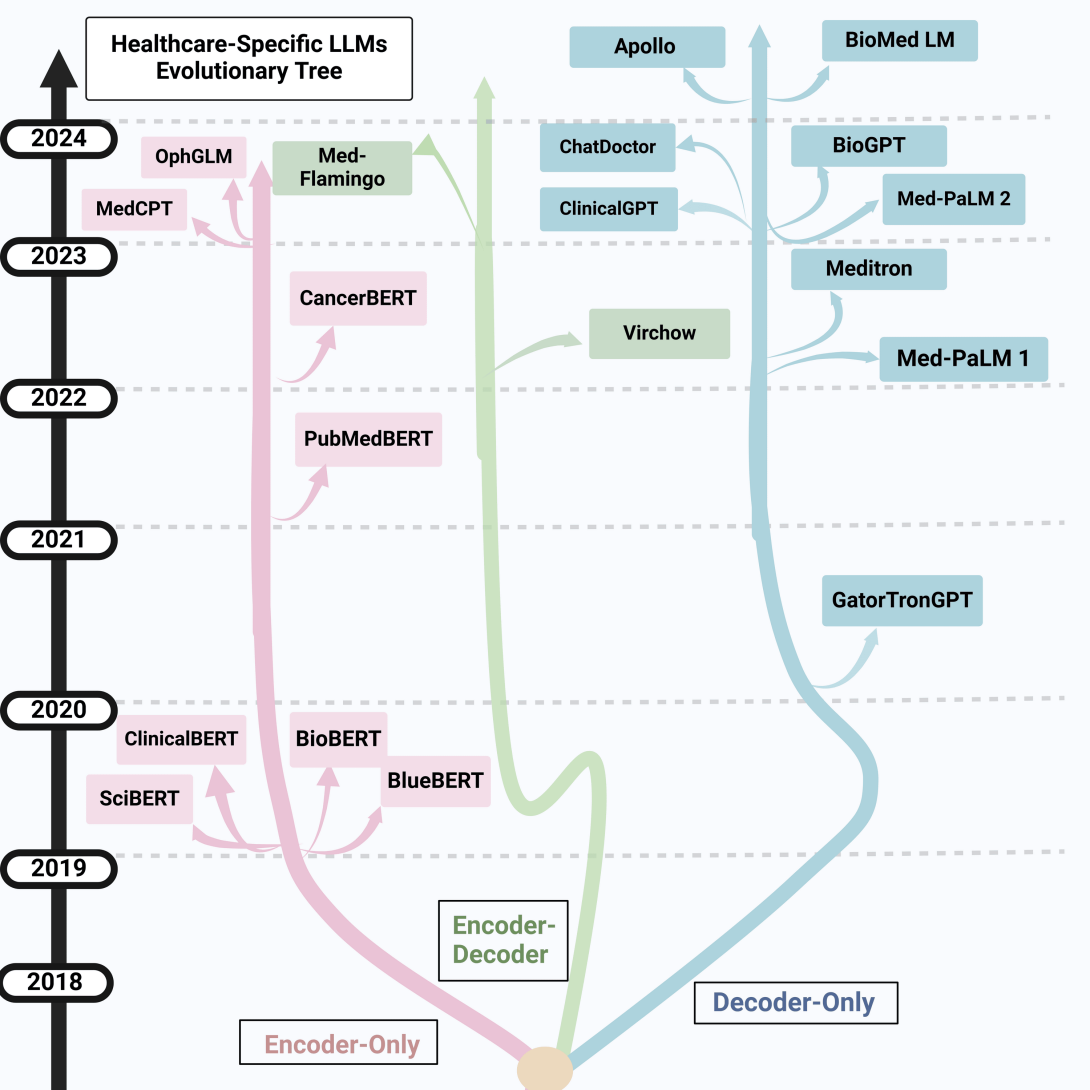
Healthcare LLM development over the years Figure credit: Julia Maslinski LLM: Large language model

Since the arrival of generative pre-trained transformer (GPT), there is increasing research work being done to understand where and how these powerful models can be applied in healthcare [7]. Over the last couple of years, there has been a rapid growth in not only generic LLMs, but also in numerous healthcare-specific LLMs trained on healthcare data, including electronic health record data (Figure 1) [8]. New frameworks for LLM implementation, such as Langchain, are being developed and refined at a rapid pace. Healthcare-specific tasks, such as summarization of clinical notes, ambient documentation of clinician-patient interaction, and patient question answering, are being developed and trialed. As the use of LLMs increases, their pitfalls and challenges in utilization, ranging from inaccuracies, hallucination generation, bias, and cost, are being realized. Increasingly, research is also focusing on understanding these issues in a better way and how to mitigate them [9].

With the excitement about the use of LLMs, large amounts of resources are being allocated for their rapid development and integration in healthcare.This will likely support and promote continued LLM research and implementation in healthcare at a rapid pace. It is important that as the number of publications related to this new and evolving technology in healthcare grows, clinicians understand the basics of LLMs to be able to read and interpret publications related to this domain. This primer aims to educate clinicians and encourage them to participate in research related to LLM applications in healthcare.

## Review

LLM basics 

LLMs are a type of AI model (Table 1) that are developed with advanced deep-learning neural network techniques. Most LLMs architecture is inspired by the transformer architecture, which consists of two key blocks: the encoder and the decoder [10]. Both the encoder and decoder are multilayered neural networks that use self-attention mechanisms and process data in a feed-forward manner. The encoder processes the data and encodes it to generate the desired output such as in classification of diseases. The decoder takes the encoded data and decodes it for the desired output such as text summarization. LLMs can be classified into three categories based on the encoder and decoder. The encoder-only models were proposed in the bidirectional encoder representations from transformers (BERT) paper and later adapted for healthcare applications with models such as BioBERT, PubMedBERT, ClinicalBERT, and others [11-14]. Models such as BERT can learn contextual representations of words and sentences, encoding the relationships between symptoms and diseases (Table 1). As in ClinicalBERT, readmission prediction can be performed by training the models on discharge summaries and physician notes in the intensive care unit. The decoder-only models include only the decoder part of the transformer, such as GPT models, including healthcare models like ClinicalGPT and BioGPT [15-17]. These models have been shown to perform well at tasks such as medical question and answering in examination scenarios [16]. The encoder-decoder models incorporate both the encoder and decoder, such as Med-Flamingo and Virchow [18,19]. Models such as Med-Flamingo can extend to generative medical question answering abilities to include multimodal data, such as a combination of images and text, to address visual question and answering in examination problems [18].

**Table 1:** Terms related to LLMs and their explanations LLM: Large language model; AI: Artificial intelligence; BERT: Bidirectional encoder representations from transformers; GPT: Generative pre-trained transformer; PLM: Pre-trained language model; RLHF: Reinforcement learning from human feedback; RM: Reward model; PPO: Proximal policy optimization; RAG: Retrieval augmented generation

Terms	Explanation
LLMs	A type of AI model trained on large amounts of data to generate language responses to user input.
Transformer Architecture	A type of neural network model that is used in natural language processing to develop LLMs.
BERT	A transformer architecture-based AI generative model that many LLMs are based on. It processes text from both directions, left-to-right and right-to left, allowing it to more efficiently grasp the context.
GPT	A transformer architecture-based AI generative model that many LLMs are based on. GPT is trained for natural language-processing tasks that can use previous words to predict the next words and form coherent sentences. It generates text sequentially rather than bidirectionally, but is usually trained on more data than BERT.
PLMs	A framework LLM that can be used in their development, as they are already trained on large data sets. PLMs can be customized to fit the uses of the specific LLM being developed.
Fine-Tuning	Training an LLM on new data and adjusting the parameters to ensure it is well-suited for all types of inputs to deliver the desired output. Training on domain-specific data allows specialization of general baseline PLMs.
RLHF	Optimization method for LLMs where the model is updated based on positive and negative human feedback to improve the model over time. This is a supervised learning method as human-labeled data is used to train the model.
RM	A part of RLHF where human-labeled data, usually a ranking of best to worst response from a sample of responses, trains the RM. This model is then used to predict which response the labeler would rank as best, which is then used as a reward function to improve the PLM.
PPO	Another part of RLHF where the RM provides a reward for the output and this reward updates the final LLM.
Prompt	User input that the LLM generates a response to, based on patterns it learned during the training process.
Prompt Engineering	An optimization method for LLMs in which the inputs are designed and formatted in a way the LLM can understand to produce a desired output.
Zero Shot	A learning method for LLMs in which they can generalize and respond to inputs they have never seen before. It is one of the key features of LLMs that make them so useful to all types of inputs and data.
Temperature	A randomness factor of LLMs that can be adjusted by the user. A low temperature means a less varied response, while a high temperature allows a more creative and diverse output to the prompt.
RAG	Optimizes LLM output by integrating data from external sources before producing a response. This allows the model to have access to current,referenced and reliable data beyond its original training set.
LLM Hallucination	The LLM generates grammatically correct and coherent sentences, but they have factually incorrect information.

LLMs are trained on extensive datasets, including text from articles, books, and websites, aiming to generate human-like responses [20]. By learning from vast amounts of data, LLMs recognize patterns and respond to human input in ways that can perform tasks such as language translation, summarization, answering questions, and text generation [21]. In contrast, traditional deep learning models use far fewer parameters than LLMs and are usually specialized for one task such as determining stroke risk based on patient information input. The recently developed LLMs have billions of parameters, are trained on vast amounts of data, utilize massive computational resources, have broad applicability, and can adapt and update responses in real-time based on user input [21]. The real-time adaptation and broad-use aspect of LLMs that are not present in other traditional deep-learning algorithms make their applicability much greater, especially for healthcare.

Development of LLMs

**Figure 2:**
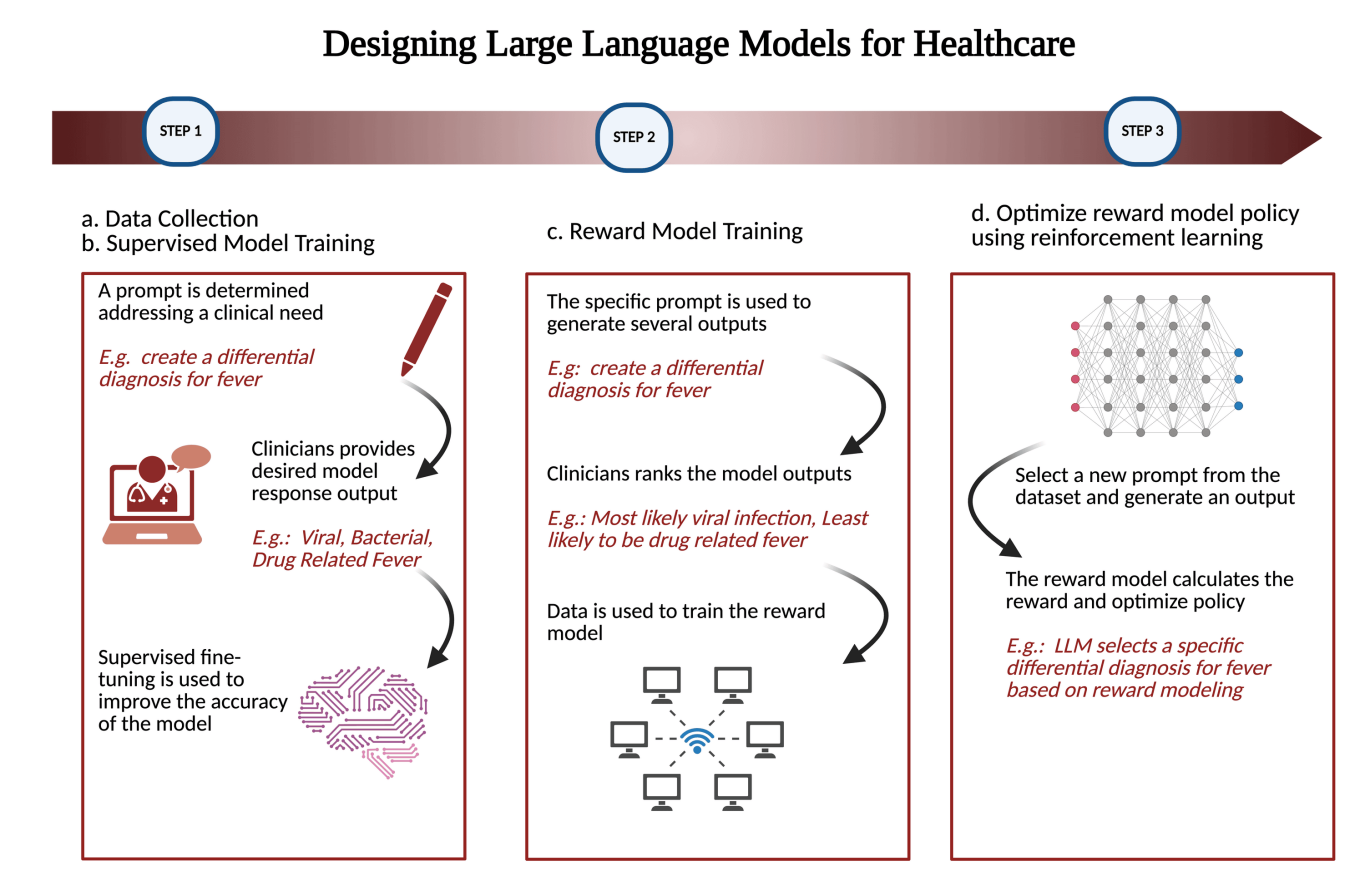
Flowchart for designing and developing healthcare-specific LLM Figure Credit: Rachel Grasfield and Raghav Awasthi LLM: Large language model

LLMs are developed using two methods, both of which have the same final steps. The first method is pre-training LLMs from a defined set of data (Figure 2). After the objective of the model is identified and an extensive amount of data is available for training, the model can be developed [22]. Next, the model is trained on the data, which may take days or weeks depending on the size of the data and computing resources.

The second method involves using existing pre-trained language models (PLMs), which is less resource-intensive as it uses an already-existing model as the base for the new LLM. With a PLM, the steps of obtaining extensive training data and selecting an architecture for the model can be skipped. To develop a model with this method, one must first find a well-suited PLM by considering both the model size and the training data that was used. It is important that the training data is diverse and useful for the model being developed. The chosen PLM should fit the intended uses of the LLM being developed. 

Once the model is developed using either of the methods previously described, validation data should be used to validate the model’s performance on data other than the training data. This step is crucial for both pre-training LLMs and using PLMs to ensure the model responds well to never-before-seen data and is able to generalize to generate responses based only on the training data it was exposed to. The model should be modified accordingly and even re-trained on more data if it is not performing to the expectations. Further, the model must be fine-tuned to ensure it is responding correctly based on user input. The most common method used for fine-tuning is reinforcement learning from human feedback (RLHF). RHLF is a supervised learning method as human-labeled data is used to train the model (Figure 2). The method involves a team of humans tasked with preferencing the responses provided by the model. For a given prompt, a human labeler will indicate the desired response, and this is used to fine-tune the model using supervised learning. Next, a given prompt and a few outputs will be sampled, for which the labeler must rank the best to worst response, which trains the reward model (RM) (Figure 2). The trained RM is then used to predict which response the labeler would rank as best. It is then used as a reward function to fine-tune the baseline model, such as proximal policy optimization (PPO), another reinforcement learning algorithm where the RM provides a reward for the output, and this reward updates the LLM [23]. RLHF is used to optimize the finalized LLM over time through positive and negative human feedback to constantly improve the model over time. 

Healthcare-specific LLM development

While many of the popular general-purpose LLMs, such as GPT, are valuable for a broad range of tasks, they are not specifically developed for use in clinical medicine. However, there are numerous emerging LLMs created specifically for clinical purposes (Figure 1). GatorTronGPT is an example of such a model that was trained on 277 billion words of mixed clinical and English text, exhibiting state-of-the-art performance in biomedical datasets [24]. GatorTronGPT was developed specifically to generate and comprehend clinical text, which is useful in medical documentation [8]. Similarly, ClinicalBERT was successful in predicting 30-day hospital readmission using only clinical notes from the first few days and discharge summaries [25]. Another example of a healthcare-specific LLM is Google’s Med-PaLM, whose performance on the United States Medical Licensing Examination (USMLE) style questions had an accuracy exceeding 67% on the MedQA dataset [2]. Med-PaLM is designed to answer medical questions with high accuracy [2]. LLMs specifically developed for healthcare tasks such as generating clinical notes, predicting readmission, and answering medical questions are extremely useful and are likely to be increasingly prevalent in clinical practice soon.

Data Type and Needs for LLMs

To successfully develop an LLM, extensive amounts of data is necessary. A diverse dataset taken from articles, websites, and books allows training of the LLM for optimal performance. The type of data necessary depends on the intended uses of the LLM being developed. If the LLM is being developed for a specific task, it should be trained and optimized with a vast amount of data from that specific topic. For example, to train a healthcare focused LLM to help perform clinical tasks such as ClinicalGPT, training on electronic health record data would be ideal [16].

To optimize the quality of the training data gathered, deduplication methods are often used. Deduplication allows sampling of only unique instances of data, so repeats are removed. Furthermore, filtering of the training data involves analysis of the data quality, which leads to removing some of the unneeded data in training the model. Finally, a remixing stage is utilized to ensure the diversity and broad applicability of the dataset. After this stage, data may be added to fill in underrepresented domains noticed during this stage. 

Scaling laws in the development of LLMs refer to the relationship between model size, amount of training data being used, and the computational resources required for the LLM to be trained. These laws suggest that the LLMs’ performance improves with increases in these three factors: model size, data amount, and computational power [26]. The larger the model size, the more likely it is able to perform complex language-related tasks. Similarly, the larger and more diverse training dataset, the better the LLM is likely to perform on a wide-range of inputs. Following this, the larger the size of the LLM, the more likely it needs more computational power to train, but in turn, leads to a better model overall. Large amounts of high-quality and diverse datasets are key to accurate LLM functioning and development, and it is what distinguishes their performance from other AI models. 

Validation and Assessment of LLM Performance

Validation and assessment of LLMs can be defined as an evaluation that checks various dimensions of output such as accuracy, fluency, relevance, bias, coherence, potential harm, and hallucinations (Table 2). Overall evaluation of LLMs can be categorized into two categories: 1) automated evaluation, which includes mathematical formulas or model-based approaches; and 2) human evaluation, where humans manually assess the LLMs' output [27].

**Table 2:** Key indicators and their corresponding metrics for evaluation [28-34] ROGUE: Recall-Oriented Understudy for Gisting Evaluation; LLMBI: Large Language Model Bias Index; HONEST: Hurtfulness of Language Model Sentence Completion; GPT: Generative pre-trained transformer

Evaluation Indicators	Quantitative Metrics	Explanation
Task Success Rate	Accuracy	Accuracy is a measure to calculate the percentage of the correct predictions by the models.
Fluency	Perplexity score	Perplexity measures the amount of randomness in the generated text by LLM.
Relevance	ROUGE score	A set of metrics used to evaluate how well a generated text such as a summary matches the reference text.
Biasness	LLMBI	LLMBI is the weight-adjusted sums of various dimensions of bias, such as gender, race, etc.
Coherence	Coh-Metrix	Coh-Metrix is an exhaustive computational tool that evaluates the cohesion of text using over 200 measures.
Hurtfulness	HONEST	HONEST is a scoring evaluation system designed to assess the harmfulness of sentence completions generated by language models.
Hallucinations	SelfCheckGPT	SelfCheckGPT is a method to detect the hallucination of LLMs.

Automated Evaluation

Automatic evaluation of natural language processing (NLP) systems, in general, is designed to be task-specific . For example, the Bilingual Evaluation Understudy (BLEU) score is more appropriate for language translation, the Recall-Oriented Understudy for Gisting Evaluation (ROGUE) score is more relevant for text summarization, and the perplexity score is used to evaluate the quality of text generation (Table 2) [35]. For text classification, confusion matrix-based scores, such as accuracy, sensitivity, specificity, and area under the receiver operating characteristic curve (AUROC), are utilized. These metrics have different interpretations. For instance, higher BLEU and ROUGE scores indicate better model performance, while lower perplexity suggests better performance [30]. However, we understand that LLMs like GPT, Large Language Model Meta AI (LLaMA), BigScience Large Open-Science Open-Access Multilingual Language Model (BLOOM), Falcon, etc., are capable of performing multiple tasks such as language translation, text summarization, sentiment analysis, and text classification [36-38]. Therefore, benchmarks like SuperGLUE, the lm-eval Python package by BIG-Bench, and MT-Benchmark, have been developed [39-41]. These benchmarks provide multiple task-specific datasets and metrics to evaluate LLMs and benchmark them. This benchmarking helps in comparing models on different tasks and making better choices of models for different use cases. Bias, leakage of private data, and toxicity are additional key factors to consider when evaluating LLMs.

Human Evaluation

Despite the development of these benchmarks and new automatic evaluation techniques, it is widely reported that while models may perform well on these benchmark datasets, they often fail to perform equally well when faced with user-specific datasets [42]. This is commonly due to the lack of information in the data on which the models are trained. For example, the instruction-tuning datasets of OpenAI models are unknown (not open-source) [43]. Also, automatic evaluations techniques are not robust to various adversarial attacks, including lexical overlap and factuality errors. Previous claims suggest that existing evaluation metrics are not robust against even simple perturbations and may disagree with scores assigned by humans to the perturbed output [44,45]. Furthermore, there is very little correlation between these metrics and human judgment [46]. Considering the challenges associated with the automatic evaluation of LLMs, human evaluation is one of the key methods to robustly assess LLMs. Human evaluation has been recently conducted in recent LLM publications and remains the gold standard method to evaluate LLM performance, particularly in fields like healthcare [47-49]. In a review of 142 publications, researchers demonstrated significant gaps in variability, generalizability and reliability of human evaluations of LLM outputs [49]. To addresses this gap, the authors have proposed QUEST, as a comprehensive and practical framework for human evaluation of LLMs [49]. Similarly, a robust set of metrics and a web application, named HumanELY, has also been recently proposed to enhance the standardization and efficacy of human evaluation [48].

Human evaluation of LLM outputs has its own set of challenges. It is resource intensive, and has a lack of guidance on issues such as: framing of the questions, number of samples needed, number of human annotators to minimize inter-annotator agreement (IAA), and who should perform the evaluation. To overcome challenges associated with human evaluation, recent research has explored how LLMs themselves can be utilized to perform human evaluation [48]. With the continuous development of diverse evaluation methods, the hope is to have more robust and reliable validation and assessment approaches for LLMs, thereby building trustworthy LLM-based systems.

Considerations for clinical implementation

Clinical

**Figure 3:**
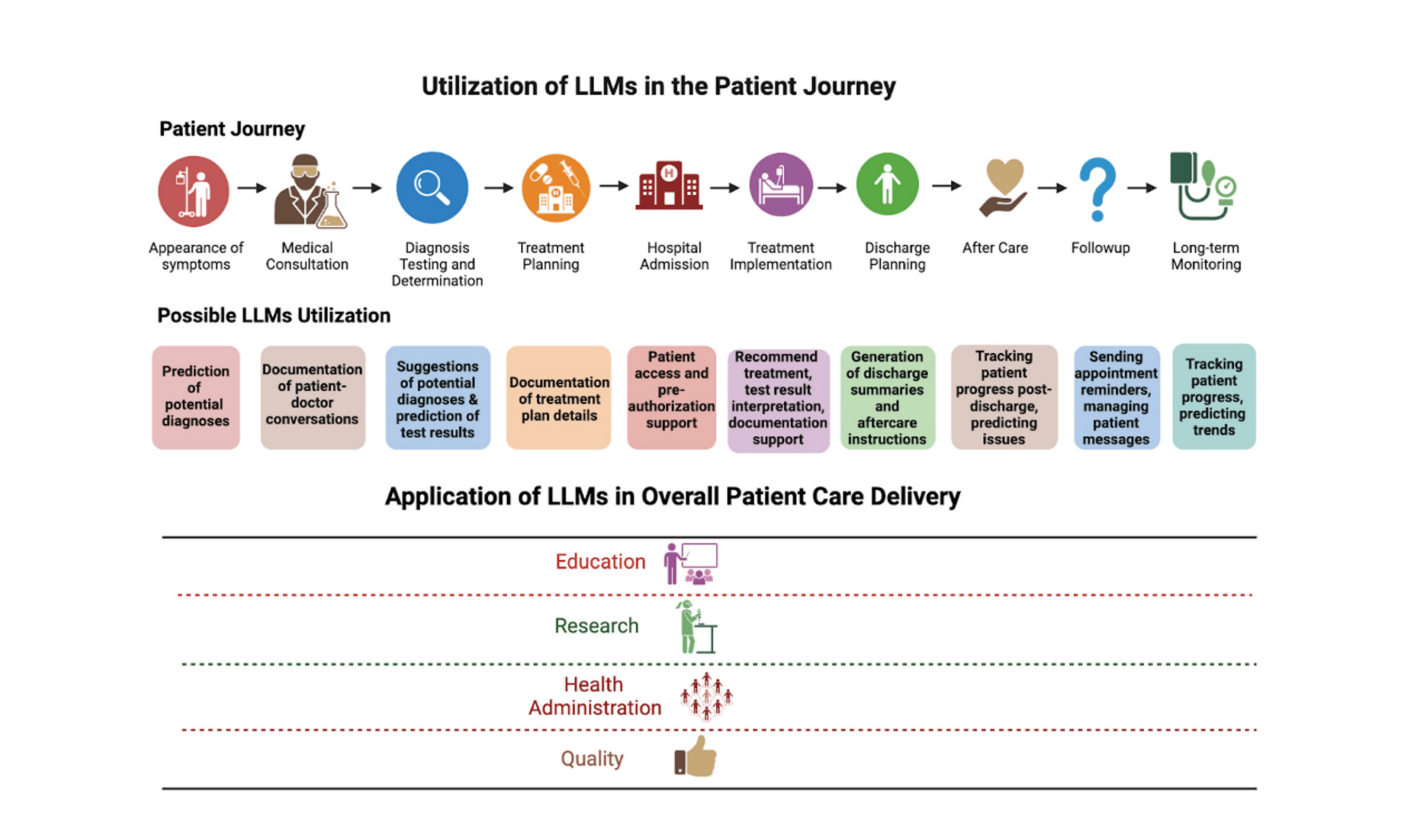
Opportunities and applications of LLMs in patient-care delivery Figure Credit: Shreya Mishra LLM: Large language model

LLMs have the capacity to be implemented in every stage of clinical diagnosis: diagnosis, management, and prognosis (Figure 3). Many studies have tried GPT to assist with diagnosis, although with variable results [50-52]. In a study of 100 patient medical records, ChatGPT was used to diagnose knee or hip arthrosis and recommend treatment plans such as pain management and surgery [53]. While the model did not perform perfectly, there was an 83% agreement between the ChatGPT and physicians on diagnosis. Treatment plans for pain approached statistical significance, and quality of life plan recommendations were statistically significant, indicating that ChatGPT could be a future diagnostic tool in analyzing patient records to prepare a diagnosis and offer treatment plans [53]. Similarly, in a study of patients with Glioma, ChatGPT responses were assessed for adjuvant therapy decision-making [54]. Experts who formed the tumor board rated the responses as ‘good’ for the ChatGPT recommendations related to treatment recommendations and therapy regimen. Similar to what the authors concluded in this study, many others have recommended a human-in-the-loop approach for complex medical decision-making with these model responses serving as useful aids [54]. LLMs have also been trialed by clinicians other than physicians for overall care delivery of patients too. For example, using ChatGPT to identify patients with type II diabetes, the model was able to suggest diet plans that matched with a nutritionists’ recommendation [ 55].

*Administrative* 

LLMs can play an important role in quickly and accurately completing various administrative tasks that are currently completed by clinicians. Clinical note documentation and summarization has been one of the key opportunities identified for use of LLM, which might help decrease clinician burnout. Use of AI scribes in some early trials has shown to be very promising as rated by physicians who used it for 10 weeks across many different specialties and many different locations [56]. Similarly, for other tasks performed by clinicians, such as writing prescriptions, ChatGPT was trained on textbook information on International Classification of Diseases (ICD) codes and rehabilitative pharmacy following a case study of a stroke [57]. It was able to accurately complete the required prescriptions and outline rehabilitation goals, as well as complete specialized therapeutic information. While only tested on a single case study, this supports a potential larger use of LLMs in writing and sending prescriptions to pharmacies, or completing medical plans and treatment goals for medical records [57].

LLMs have the ability to improve clinician functioning by maximizing the area of medicine they find most meaningful. They may be a useful tool in relieving some of this stress by providing compassionate, empathetic responses to medical questions from patients, which physicians can then review before sending. In one study, the AI Chatbot responding to patient messages was found to be significantly higher quality regarding empathy (“bedside manner”) than the physician [58].

LLMs' role in education has been one of the key focus areas in the early trials, especially using GPT. From medical school education to training specialists such as neurosurgeons and nursing education, many early studies have tried LLMs [59-61]. Many of the early studies have also compared the performance of GPT on various medical examinations, sometimes even correlating them with clinician performance [62,63]. These are early explorations in the use of LLMs in healthcare, which, over the next few years, are likely to be continuously experimented upon and will have the use cases defined and refined.

Research in medicine is very labor-intensive and inefficient. Many early use cases of LLMs in the same have been recently explored including, evidence synthesis, scientific writing, and patient record matching for clinical trial enrollment, amongst many others [64-67]. Interestingly, while many scientific journals have restricted the use of LLMs for scientific writing, the New England Journal of Medicine (NEJM) AI has allowed their use as long as authors take complete responsibility for the content and provide transparency [5]. Use of LLMs in research is a very promising area that is likely to bring in efficiencies at scale.

Patient Use

Several studies to date have demonstrated the ability of ChatGPT to serve as a bridge between physicians and patients by providing tailored responses and educational materials that best fit the patient’s level of medical literacy [68-70]. This provides a potential resource for both parties, as LLMs can serve a supportive role in providing patients with useful resources and answers to their questions, while also allowing physicians to communicate their understanding of diagnoses, prognoses, and commonly asked questions in a way that maximizes patient understanding. 

ChatGPT has demonstrated that it can provide accurate and appropriate answers to the questions about sleep apnea from a patient [69]. Prompts to ChatGPT can decrease the response grade level, improving readability while providing an acceptable level of medical information [69]. This supports the theory that ChatGPT can improve medical literacy in patients by providing accurate responses to their medical questions within the frame of educational answers that best fit their needs [69]. An area of medical education that may not be accessible to the general public is the information provided in medical journals, which may surpass the reading level or comfort of patients. For example, in the field of urology, ChatGPT has been shown to be able to read and analyze academic papers and provide a summary that fits the patient’s level of understanding [70]. While promising, limitations such as hallucinations, accuracy, and readability, of such applications still needs further evaluation [49].

Implementing LLMs in clinical practice

While studies have shown the potential benefits of LLM implementation as a clinical decision-making tool in a research framework, the reality of actual implementation may pose more challenges. Implementation of LLMs in healthcare facilities requires a strategy around managing the following key aspects of deployment: 1) data storage, 2) LLM deployment, and 3) healthcare professional interface. Data storage, especially of electronic medical record (EMR) data, for most hospitals is managed using on-premise servers or cloud services [71]. LLM deployment can occur on a server/cloud within the EMR or externally, which interacts with the stored data. Delivery of LLM output is most likely to occur through EMRs or other devices including mobile. All these require significant additional resources, including bandwidth for data transmission, cybersecurity applications, and trained professionals for managing them.

Additionally, multidisciplinary teams that can guide the implementation strategy, including ethical concerns, legal issues, and resource allocation, similar to other AI model deployments, are important [72]. The type of information stored, both onsite or in the cloud, may contain sensitive and identifying information. Patient healthcare data is protected by medical professionals and regulators using the Health Insurance Portability and Accountability Act (HIPAA) standards. This is a significant issue, considering that chatbots are not immune to data breaches and leaks, and have the capacity to share personal information with those who can manipulate the algorithm. Additionally, chatbots have been theorized to evolve such that physicians can be manipulated into including patient information in their queries, which may be sold to third parties [73].

Measuring and Monitoring Clinical Effect

The purpose of deploying LLMs in a clinical environment is to support clinician decision-making to deliver high-quality care. Measuring the performance of LLMs, both on technical and clinical benchmarks, is essential. While the field of LLM operations (LLMOps) is nascent, it is important to incorporate it in the strategy for LLM deployments [74]. Establishing mechanisms for monitoring model and pipeline lineage, with alerts for detecting model drift and identifying potential malicious user behavior, is critical from a technical point of view. From a clinical point of view, establishing key clinical metrics for clinical decision support delivery, adoption, and feedback need to be established.

Evaluating and Interpreting LLM-Based Publications and Contextualizing the Results

Similar to any other AI model, training data forms the basis of the likely output and performance of the model. One of the key aspects of understanding research related to LLMs is to understand the data they are trained on. There are many models that are trained on generic Internet-extracted data, which may or may not perform well on specific healthcare tasks. LLMs trained on PubMed, patient-clinician interaction data, or electronic health record data are likely to exhibit varying performance across different tasks.

It is also important to understand the differences in performances of these LLMs for different technical tasks such as data summarization, question answering, and data classification. Based on the training data and model architecture, LLMs might behave differently for these tasks. It is important to understand the base performance of these using task-based evaluation metrics, the appropriateness of their use, or alternatives. Many of these models are not static either, and their versions are updated with new data or learning architecture.

Most of the models researched in healthcare publications so far have been used “out of the box” with limited prompt engineering. It is important to understand if any significant modifications were made or can be made to impact model output, such as fine-tuning which might change the performance from that of the base model. Further, determining how these models have been used by including techniques like retrieval augmented generation (RAG) is important.

Understanding the evaluation and performance metrics used in researching these LLMs is crucial. While technical metrics might be helpful, comprehensive and standardized evaluation methods, including those for human evaluation, are also important. Ultimately, understanding the performance of these LLMs in healthcare applications is necessary. While many have compared the LLM with human performance, understanding the goals of the research and the impact on patient care delivery is key. LLMs might outperform or underperform in some areas of healthcare, but these outcomes must be interpreted cautiously, in the context of precision care delivery and the generalizability of results across various patient populations. 

To ensure transparency and standardization in reporting of LLM model for individual prognosis or diagnosis, Transparent Reporting of a Multivariable Prediction Model for Individual Prognosis or Diagnosis-LLM (TRIPOD-LLM) is one of the first guideline with a checklist of 19 main items and 50 subitems [75]. This guideline builds on the prior reporting statement of TRIPOD + AI [75]. Evaluation frameworks, such as HumanELY and QUEST, also provide additional checklists that can be used to report human evaluation of LLM model outputs [48,49]. Standardization of methodology in research using evaluation frameworks and reporting guidelines are likely to improve the quality of publications related to LLM in healthcare in the future.

## Conclusions

Availability of state-of-the-art AI models, such as LLMs, is very exciting and spurs large-scale research for their application in healthcare. These models provide us with an unprecedented opportunity to improve all aspects of healthcare, including clinical and administrative tasks. It is important for clinicians to understand the basics of these models as well as their adoption methods and pitfalls to be able to interpret this important research work and related publications.
